# Modeling Long-Term Host Cell-*Giardia lamblia* Interactions in an *In Vitro* Co-Culture System

**DOI:** 10.1371/journal.pone.0081104

**Published:** 2013-12-03

**Authors:** Bridget S. Fisher, Carlos E. Estraño, Judith A. Cole

**Affiliations:** Department of Biological Sciences, University of Memphis, Memphis, Tennessee, United States of America; Centro de Investigacion y de Estudios Avanzados del Instituto Politecnico Nacional, Mexico

## Abstract

Globally, there are greater than 700,000 deaths per year associated with diarrheal disease. The flagellated intestinal parasite, *Giardia lamblia*, is one of the most common intestinal pathogens in both humans and animals throughout the world. While attached to the gastrointestinal epithelium, *Giardia* induces epithelial cell apoptosis, disrupts tight junctions, and increases intestinal permeability. The underlying cellular and molecular mechanisms of giardiasis, including the role lamina propria immune cells, such as macrophages, play in parasite control or clearance are poorly understood. Thus far, one of the major obstacles in ascertaining the mechanisms of *Giardia* pathology is the lack of a functionally relevant model for the long-term study of the parasite *in vitro*. Here we report on the development of an *in vitro* co-culture model which maintains the basolateral-apical architecture of the small intestine and allows for long-term survival of the parasite. Using transwell inserts, Caco-2 intestinal epithelial cells and IC-21 macrophages are co-cultured in the presence of *Giardia* trophozoites. Using the developed model, we show that *Giardia* trophozoites survive over 21 days and proliferate in a combination media of Caco-2 cell and *Giardia* medium. *Giardia* induces apoptosis of epithelial cells through caspase-3 activation and macrophages do not abrogate this response. Additionally, macrophages induce Caco-2 cells to secrete the pro-inflammatory cytokines, GRO and IL-8, a response abolished by *Giardia* indicating parasite induced suppression of the host immune response. The co-culture model provides additional complexity and information when compared to a single-cell model. This model will be a valuable tool for answering long-standing questions on host-parasite biology that may lead to discovery of new therapeutic interventions.

## Introduction

*Giardia lamblia*, also known as *G. intestinalis* or *G. duodenalis*, is one of the most common intestinal protozoan parasites in humans, wildlife, and domestic animals. Primarily transmitted through drinking water contaminated with parasite cysts [1] more than one billion people were at risk for contracting *Giardia* in 2000 [2]. Infections are particularly high in developing countries with inadequate drinking facilities [3], in child care centers [4], and in immunocompromised individuals [5]. Due to its global distribution and significance, in September 2004, the World Health Organization (WHO) included *Giardia lamblia* on its ‘Neglected Disease Initiative’ in an effort to resolve long-standing questions on parasite biology, epidemiology, treatment, and host-parasite interactions [6], [1].

A hallmark feature of *Giardia* infections is the wide range of symptom presentation. The majority of infected individuals exhibit few signs and symptoms of infection. Other hosts display abdominal cramping, nausea, bloating, weight loss, vomiting, malabsorption, and acute or chronic diarrhea (Reviewed in [7]). Despite the clinical variation of giardiasis in active trophozoite infections, giardiasis does not cause overt inflammation of the intestinal epithelium [8] except in cases of prolonged disease [9]. Much work has been done to identify the underlying cause(s) of symptom variation, including parasite load [10], *Giardia* assemblage associated with the infection [11], [12], antigenic variation in the parasite [13], infectious dose [14], and host immune status [9]. Currently, it is thought that a multitude of factors lead to the clinical manifestations of the disease.

Although the symptoms associated with *Giardia* infection have been well documented, the underlying cellular and molecular mechanisms leading to disease are not well understood. Epithelial cells exposed to *Giardia* exhibit increased expression of stress response genes, decreased proliferative gene expression [15], actin rearrangement [16], tight junction disruption [17], increased intestinal permeability [18], [17], and apoptosis [19], [18]. Additionally, when exposed to the parasite *in vitro*, epithelial cells secrete cytokines that are chemotatic for immune cells, including macrophages [15]. Recruitment of macrophages to the site of infection suggests that these cells have a function during parasite control and/or clearance. Mice infected with *Giardia muris* exhibit decreased recruitment of inflammatory cells to the peritoneal cavity and macrophages isolated from infected animals have reduced chemotatic responsiveness [20], but retain the ability to phagocytose trophozoites [21]–[23]. However, in human giardiasis, it is unclear how macrophages respond to cytokines secreted from epithelial cells during infection and subsequently modulate the host immune response.

Most of the studies modeling *Giardia*-host interactions have involved animal models and *in vitro* monolayer co-culture experiments. In addition to the cost and ethical issues involved in employing animal models, the species specificity of *Giardia lamblia* makes animal studies problematic. Studies utilizing *Giardia muris* to infect mice are not likely to accurately represent human giardiasis as both a different host and *Giardia* species are used [17] and there are important differences between mouse and human immunity (reviewed in [24]). Additionally, different mouse strains have yielded disparities in parasite control and clearance [25], [26] and colonization of the parasite is dependent on the gut microflora of the host, which can differ between laboratories [27]. Together these data indicate that the mechanisms of immune control during giardiasis may differ substantially between humans and mice.

*In vitro* cell models have yielded valuable insight into disease pathology, immune-regulatory mechanisms, and underlying signaling pathways; however, these monolayer co-culture assays do not accurately reflect the three-dimensional nature of the gastrointestinal tract and the complex intra-cellular communication in host tissue [28]. To further expand the knowledge of cellular signaling and immune mechanisms during a *Giardia* infection, a better understanding of cell-cell interaction in the gut during infection is paramount. We developed a co-culture system of the gastrointestinal tract which serves as an intermediate between simplistic monolayer co-culture *in vitro* studies and dynamic *in vivo* biological processes (Reviewed in [29]). This model will facilitate the understanding of cell-cell interactions during infection and the variability of symptoms associated with giardiasis in the host.

## Materials and Methods

### Cell culture

A human colonic adenocarcinoma cell line, Caco-2 cell clone C2BBe1 [30], was obtained from the American Type Culture Collection (CRL-2102). Caco-2 cells (passages 57–72) were cultured at 37°C, 5% CO2 in Dulbecco's Modified Eagle's medium (DMEM; Cellgro, Manassas, VA) supplemented with 10% fetal bovine serum (FBS) (Life Technologies, Grand Island, NY), 100 U/ml penicillin, and 100 µg/ml streptomycin. Cells were feed every third day and passed using 0.025% trypsin with 0.22 mM EDTA when ∼80–90% confluent. IC-21 cells [31], a murine peritoneal macrophage cell line, were obtained from Dr. Richard Smith (University of Tennessee Health Science Center, Memphis, TN). IC-21 cells were maintained in RPMI-1640 supplemented L-glutamine (Cellgro, Manassas, VA), 10% FBS, 100 U/ml penicillin, and 100 µg/ml streptomycin. Cells were feed every other day and passed with Hanks Balanced Salt Solution (HBSS) when confluent.

### Parasites

*Giardia lamblia* strain WB clone C6 was obtained from the American Type Culture Collection (#50803). Parasites were grown in filter sterilized modified TYI-S-33 medium with 10% adult bovine serum and 0.05% bovine bile at 37°C in microaerophilic conditions and subcultured when confluent [32]. To collect parasites for experiments, the medium was removed from the culture to eliminate unattached or dead parasites. The tube was refilled with cold, sterile medium and trophozoites detached by chilling on ice for 15 minutes. Parasites were collected by centrifugation (1500 x g for 5 minutes at 4 °C) and washed once with the plating medium of 90% complete DMEM/10% *Giardia* medium. Parasites were then counted using a hemocytometer and diluted to the appropriate number.

### Co-culture model

**Figure 1:**
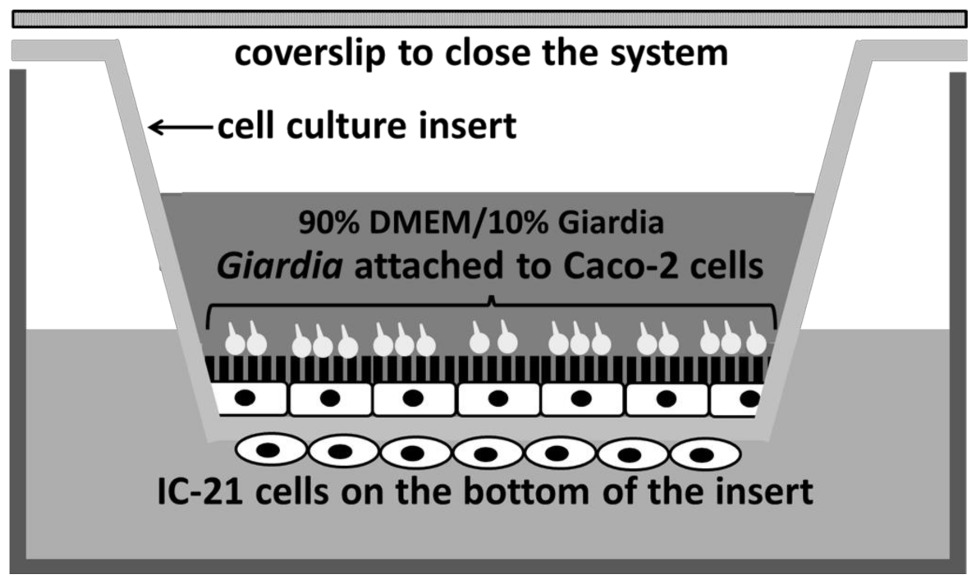
Co-culture experimental design. Caco-2 cells are grown on the upper surface of 24-well inserts in complete DMEM. IC-21 macrophages are plated at on the bottom of the inserts in complete RPMI media. After 72 hours, *Giardia* trophozoites are added to the inserts in 90% complete DMEM with 10% *Giardia* media. Control inserts receive only the combination medium. Inserts are filled to the top with media and sealed with a round cover-slip.

IC-21 cells were plated on the bottom of a 0.4 µm translucent polyethylene terephthalate (PET) membrane cell culture insert (BD Falcon, San Jose, California, catalog number 353095) in complete RPMI (RPMI supplemented with FBS, 100 U/ml penicillin, and 100 µg/ml streptomycin) at 500,000 cells/ml. Briefly, 3 inserts were placed upside down per well containing 1 ml HBSS in a 6 well plate and the bottom of the insert coated with 60 µl of the cell suspension. The macrophage coated inserts were covered with the plate lid, and allowed to adhere overnight at 37°C and 5% CO2. The next day, each insert was placed in 800 µl medium (Figure 1) in a 24-well plate (Corning Life Sciences, Corning, NY, catalog number 3524). The top of the insert was seeded with 250 µl Caco-2 cells at 600,000 cells/ml in complete DMEM (DMEM supplemented with FBS, 100 U/ml penicillin, and 100 µg/ml streptomycin) (Figure 1). The co-culture was incubated at 37°C and 5% CO2 for 72 hours, then the Caco-2 cell media was removed and replenished with a combination of 90% complete DMEM/10% *Giardia* medium plus or minus *Giardia* trophozoites (100,000 total parasites/insert). All wells were filled to the top with additional media and then sealed with a round cover-slip and incubated at 37°C and 5% CO2 (Supplemental data, Video S1). For the first 5 days, cells were fed by removing half of the media (∼500 µl) and adding fresh media to the well and insert every other day. After 5 days, unattached parasites were eliminated by completely removing all the media and replenishing with fresh media every day. Control co-cultures were maintained in a separate plate to prevent parasite contamination. Control inserts were inspected daily under the microscope to ensure there was no *Giardia* cross contamination. Once incubation time was complete, parasites were removed using 10 µM formononetin as described previously [33]. Briefly, the culture media was removed from the insert and replenished with 1 ml of 10 µM formononetin in DMEM. Inserts were incubated at 37°C for 5 minutes and then gently washed three times with PBS.

### Effect of formononetin treatment on signaling in Caco-2 cells

Lauwaet et al [33] used the isoflavone formononetin to rapidly detach *Giardia* from intestinal cells. Prior to utilizing this method, its effect on Caco-2 cell pro-apoptotic and proliferative signaling was assessed. Caco-2 cells were plated at 60,000 cells/ml in 96-well plates and cultured for 3 days to confluence. Formononetin (Acros Organics/ThermoFisher, Waltham, MA) was added at 10 µM and 40 µM final concentrations for 5 minutes. The Caco-2 cell layer was washed 3 times with PBS and the effect of formononetin treatment on MAPK signaling involved in proliferation (ERK) and stress responses (p38 and JNK) were assessed using the PACE assay as described below. The data of four experiments assayed in triplicate are reported as percent change over control (Caco-2 cells not exposed to formononetin) ± SEM.

### Phosphospecific antibody cell-based ELISA (PACE)

MAPK activity was measured as described in [34]. Briefly, cells were treated as described in each figure legend, then washed once with cold PBS and fixed in 4% formaldehyde. Endogenous peroxides were quenched with 0.6% hydrogen peroxide, and plates blocked for 1 hour in either 5% milk in PBS-0.1% Triton X-100 (PBS-T) for ERK measurements or 5% BSA in PBS-T for p38 and JNK measurements. Cells were incubated at 4°C overnight in primary antibodies [anti-phospho-p44/42 MAPK (Erk1/2) (Thr202/Tyr204) rabbit monoclonal antibody (1∶8,000), anti-phospho-p38 MAPK (Thr180/Tyr182) mouse monoclonal antibody (1∶1000) or anti-phospho-SAPK/JNK (Thr183/Tyr185) mouse monoclonal antibody (1∶1,000); Cell Signaling Technology, Danvers, MA]. Assays were developed using a peroxidase-conjugated goat anti-rabbit antibody for ERK (1∶1,000) and peroxidase-conjugated goat anti-mouse for p38 (1∶1,000) and JNK (1∶500), and 1-Step Ultra-TMB ELISA (Fisher Scientific). The reaction was stopped with 2 M H2SO4 and the absorbance was read at 450 nm using a Bio-tek Instruments ELX 808 Ultra Microplate Reader (Winooski, VT). The experimental values were compared to basal values and plotted using GraphPad Prism software (La Jolla, CA). Positive controls for ERK signaling were obtained by stimulating cells with epidermal growth factor (EGF) (PeproTech, Rocky Hill, NJ) (100 ng/ml) for 15 minutes while positive controls for p38 and JNK were obtained by incubating cells with D-sorbitol (Sigma-Aldrich, St. Louis, MO) (300 mM) for 15 minutes.

### Microscopy

Live images of the co-culture were obtained for day 1, 5, 13, and 21 using a Nikon Eclipse TS100 microscope with the Nikon Digital Sight camera. At 5, 13, and 21 days, the Caco-2 monolayer on the both the control and parasite exposed inserts was stained with crystal violet. Briefly, following *Giardia* removal, the cell monolayer was fixed in 4% formaldehyde for 30 minutes. The monolayer was gently washed three times with PBS and then stained for 30 minutes in 4% crystal violet. The stained monolayer was decolorized in water and then imaged. Images of *Giardia* trophozoites removed from the model were also obtained.

### Caco-2 cell activation in media mixes

Caco-2 cells plated at 60,000 cells/ml were grown on 96-well plates for 3 days to reach confluence in 100% complete DMEM. The medium was then removed and different media mixes composed of 100%/0%, 90%/10%, and 75%/25% complete DMEM to *Giardia* medium were added. Changes in signaling were assessed 24 and 48 hours later using phosphospecific antibody cell-based ELISAs (PACE) as described above. Data are reported as the fold change in signal when compared to the control of 100% DMEM ± SEM assayed four times in triplicate.

### Caco-2 cell viability in media mixes

Caco-2 cells were plated at 60,000 cells/ml in 24-well plates and cultured to confluence in 100% complete DMEM. The medium was then replaced with the different media mixes and cell viability was assessed over the course of 7 days using trypan blue staining. Briefly, the cell monolayer was treated with trypsin to obtain individual cells. The cells were collected by centrifugation and resuspended in 500 µl warm PBS with 0.04% trypan blue. Cells were immediately imaged and the number of trypan blue positive cells were compared to the total number of cells to obtain the percent viability. Each replicate was imaged three times and the average calculated. Values are reported as the percent change when compared to 100% DMEM ± SD. For morphology assessment, Caco-2 cells were plated on 8-chamber glass culture slides (BD Falcon) at 60,000 cells/ml and grown until confluent (∼5 days) in 100% complete DMEM. The medium was then removed and replenished with the different media mixes and microscopy images obtained at 1, 2, and 5 days.

### *Giardia* viability in 90/10 media mix

Caco-2 cells were seeded in a 24-well plate at 100,000 cells/ml in complete DMEM. Once confluent, *Giardia* trophozoites were added at 50,000, 100,000, and 300,000 parasites/cm^2^ in 90% complete DMEM/10% *Giardia* medium. At 24 and 48 hours, images were obtained a Nikon Eclipse TS100 microscope. Parasites were detached from the Caco-2 cells by adding ice cold PBS to the well and incubating on ice for 10 minutes. Parasites were collected by centrifugation and then counted using a hemocytometer. Data are reported as the number of parasites per square centimeter as determined by three counts each of three independent replicates.

### Cell number assay

Co-cultures were plated as previously described. *Giardia* trophozoites, added at 100,000 parasites/insert, were incubated in the system for 5 days and removed using 10 µM formononetin for 5 minutes. The Caco-2 cell layer was gently washed 3 times with PBS and cell number was assessed using a modified methylene blue assay [35]. Briefly, cells were incubated with 100 µl methylene blue solution (4% formaldehyde/0.6% methylene blue in HBSS) for 60 minutes at 37°C. After 3 rinses with deionized water, the inserts were air-dried. Color was eluted by adding 200 µl elution buffer (50% ethanol, 1% acetic acid in PBS) and incubating the plate for 15 minutes on a plate rotator. The eluted color was transferred to a 96-well plate and absorbance was read at 595 nm. A standard curve was established by plating Caco-2 cells in 24-well plate (0.25–3×10^5^), incubating at 37°C for 5 hours to allow cells to attach to plate then staining with methylene blue. Absorbance values of experimental inserts (Caco-2 with *Giardia*) were compared to control inserts (Caco-2 cells alone) and plotted with the SEM of three experiments assayed in duplicate.

### Caspase-3 assay

Caco-2 cells were seeded at 600,000 cells/ml on 6-well 0.4 µm PET culture inserts (BD Falcon) and on 6-well plates (BD Falcon, San Jose, California) using 2 ml of cells in 100% complete DMEM. The cells were incubated at 37°C, 5% CO2 for 3 days. Medium was then removed and replenished with 90% complete DMEM/10% *Giardia* medium alone or with media mix with trophozoites at 350,000 parasites/cm^2^ in both inserts and plates. Wells and inserts were filled to the top with the media mix. Inserts were sealed using sterilized transparency film and incubated for 24 hours or 5 days. Camptothecin (5 µM) was added to Caco-2 cells on both inserts and plates for 24 hours at 37°C as a positive control for apoptosis. Apoptosis was measured using the caspase-3 assay kit (ab39401) (Abcam, Cambridge, MA) according to the manufacturer's instructions. Briefly, 50 µl of 2X Reaction buffer containing 10 mM DTT was added to the sample along with 5 µl of 4 mM DEVD-p-NA substrate. The reaction was incubated at 37°C for 1 hour then the absorbance was read at 405 nm. The protein concentration of each sample was determined using a Bradford Assay and absorbance values were normalized to the amount of protein in each reaction. Values are expressed as the fold change over control ± SEM of three experiments assayed in duplicate.

### Cytokine array

Human macrophages were differentiated from blood monocytes isolated from donated buffy coats (Key Biologics, Memphis, TN) using Percoll density gradients as described [36]. Briefly, stock isotonic Percoll was prepared by mixing 9 parts pure Percoll and 1 part 1.5 M NaCl. Two Percoll concentrations, 57% and 67% (v/v), were prepared in HBSS. In 15 ml tubes, 5 ml of 57% Percoll was carefully layered over 5 ml of 67% Percoll. Buffy coats were diluted in an equal volume of calcium/magnesium-free PBS and then 5 ml added to the top of the Percoll gradient. Samples were centrifuged at room temperature at 400 x g for 1 hour. Mononuclear cells, located below the plasma band and above the 57% Percoll region, were carefully transferred to a new 15 ml tube and collected by centrifugation. The pellet was washed 2 times with HBSS, resuspended in RPMI supplemented with L-glutamine (Cellgro, Manassas, VA), 10% FBS, 100 U/ml penicillin, and 100 µg/ml streptomycin and plated in 25 cm^2^ flasks. Macrophages were differentiated from blood monocytes as previously described [37] and used to build the co-culture model described above. After 5 days of incubation with *Giardia* trophozoites, the conditioned media from Caco-2 cells and macrophages were collected. The secreted cytokines were detected using the RayBio® Human Cytokine Antibody Array (AAH-CYT-1-2) according to the manufacturer's instructions. Membranes were exposed to Classic Blue autoradiography film X (Molecular Technologies, St. Louis, MO) and the intensities of signals were quantified by densitometry using ImageJ version 1.46 for Windows (NIH Bethesda, MD, http://rsb.info.nih.gov/ij/). The intensities were normalized to the positive control on each membrane and reported as relative expression levels for the exposure time.

### Immunofluorescence analysis of culture-induced encystation

Caco-2 cells were grown to confluence in 24-well plates in 100% complete DMEM. *Giardia* trophozoites (50,000 parasites/cm^2^) were added to wells with and without Caco-2 cells in three media mixes, 100% DMEM, 90% DMEM/10% *Giardia* media, and 75% DMEM/25% *Giardia* media. Trophozoites were incubated for 24 hours at 37°C, 5% carbon dioxide. Trophozoites were removed from the wells and allowed by adhere to coverslips pre-coated with poly-L lysine at 37°C for 30 minutes. The adherent parasites were washed twice with PBS and then fixed for 5 minutes at −20°C in methanol: acetone (1∶1) followed by three washes with PBS. Cells were permeabilized in 0.1% Triton-X in PBS (PBS-T) for 15 minutes at room temperature and then blocked for 30 minutes in 5% normal goat serum in PBS-T at room temperature. Cells were incubated for 1 hour at room temperature in primary antibody for *Giardia* cysts, *Giardia* lamblia 65 kDa Antigen Antibody (Thermo Scientific Pierce Antibodies, Rockford, IL) (catalog number MA1-7437) (1∶10) in 5% normal goat serum in PBS-T. Cells were washed three times for 5 minutes each in PBS and then incubated with secondary antibody, goat anti-mouse AlexFluor 488 (Life Technologies/Invitrogen, Grand Island, NY) (1∶250) in 5% normal goat serum in PBS-T for 30 minutes at room temperature. Cells were washed three times in PBS for 5 minutes at room temperature and then mounted on clean glass slides using SlowFade® Gold Reagent with DAPI (Life Technologies/Invitrogen, Grand Island, NY). Slides were cured overnight at room temperature in darkness and then imaged using a Nikon Eclipse E800. *Giardia* cysts were generated as previously described [38] and used as a positive control for the primary antibody while trophozoites served as a negative control.

### Statistical analysis

The data are presented as means ± SEM or means ± SD as indicated in each figure legend. Nonparametric one-way analysis of variance (ANOVA), followed by Bonferroni's multiple comparison test, were used to evaluate the differences among experimental data. A *P* value of <0.05 was considered significant.

## Results

### Defining the co-culture requirements for *Giardia* and Caco-2 cells

The life cycle of the *Giardia* parasite has two distinct stages, the infective cyst and the metabolically active trophozoite found in the intestine. The full parasite life cycle can be completed *in vitro*. Cholesterol [39] and bile [38] concentrations seem to be important for controlling the parasite progression from trophozoites to cysts *in vitro*. Caco-2 cells, on the other hand, are sensitive to osmolarity of growth medium. Osmotic stress-induced changes in tight junctions and cytokine secretion are both mediated through Mitogen-Activated Protein Kinases (MAPKs) [40], [41], highly conserved protein kinases that function in vital cellular processes including cell cycle regulation, cell differentiation and proliferation, immune responses, and cell death. The three major MAPKs are extracellular signal-regulated kinase (ERK-1/2), which functions in differentiation and proliferation, the c-Jun N-terminal kinases (JNKs), and the stress-activated protein kinase (p38), which are involved in cellular response to stress. To optimize culture conditions that prevent *Giardia* cyst formation or trophozoite death while maintaining cellular characteristics of Caco-2 cells, different combinations of Caco-2 medium (complete DMEM) and *Giardia* medium were assessed with regards to MAPK signaling and Caco-2 cellular viability and morphology.

**Figure 2:**
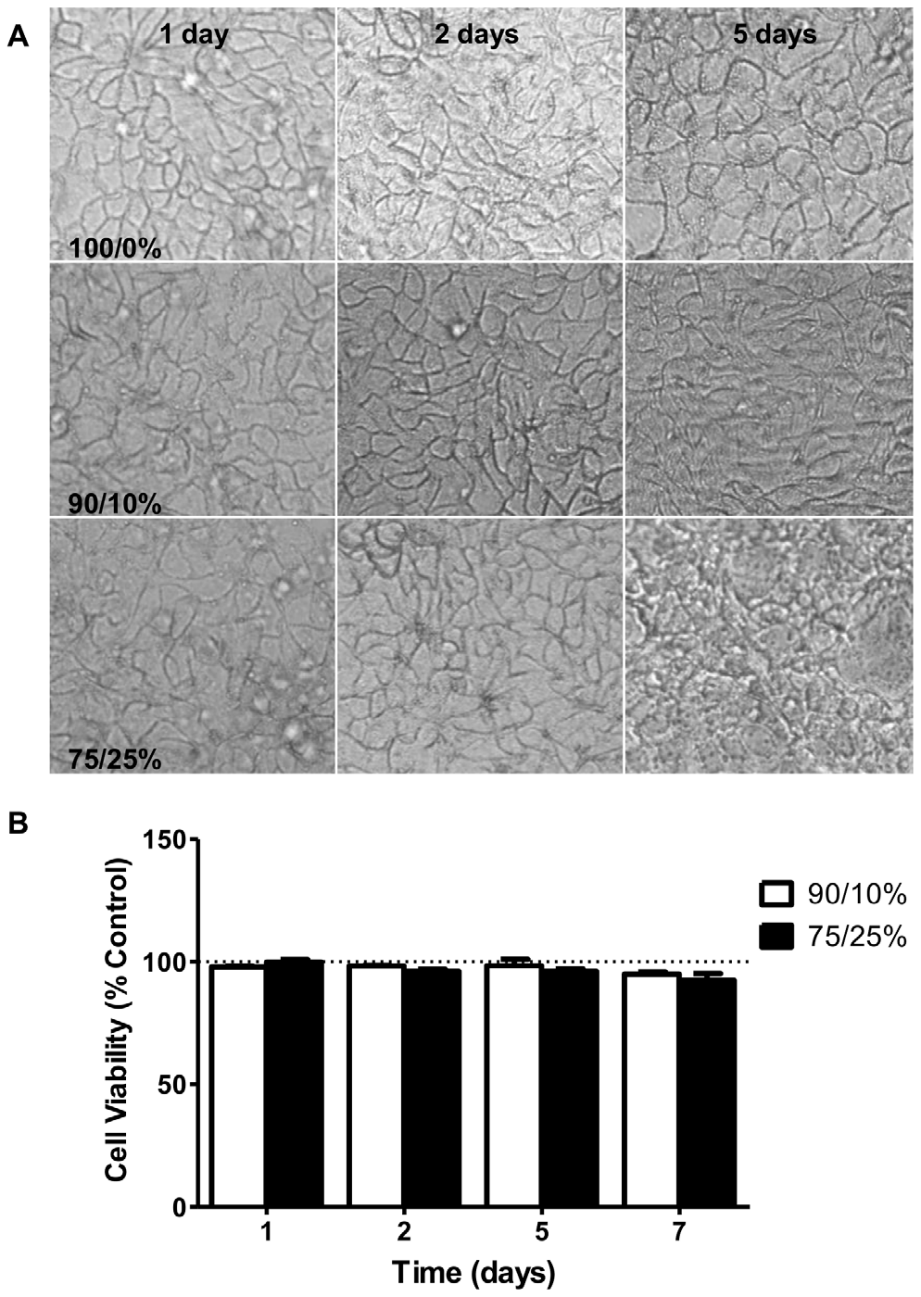
Morphology and viability of Caco-2 cells in media mixes. **A**) Images of Caco-2 cells grown to confluence in 8-chamber slides and cultured for 1, 2, and 5 days in 100% DMEM, 90% DMEM/10% *Giardia* media, or 75% DMEM/25% *Giardia* media. **B**) Caco-2 cells grown to confluence in 24-well plates and cultured with the media mixes, 100% DMEM, 90/10%, or 75/25%, for 1, 2, 5, and 7 days. Cell viability was determined using trypan blue exclusion with the number of blue cells compared to the total cell number to obtain percent viable. The data are represented as the percent change of experimental values when compared to the time-matched 100% DMEM culture conditions ± SD. (n = 1).

**Figure 3:**
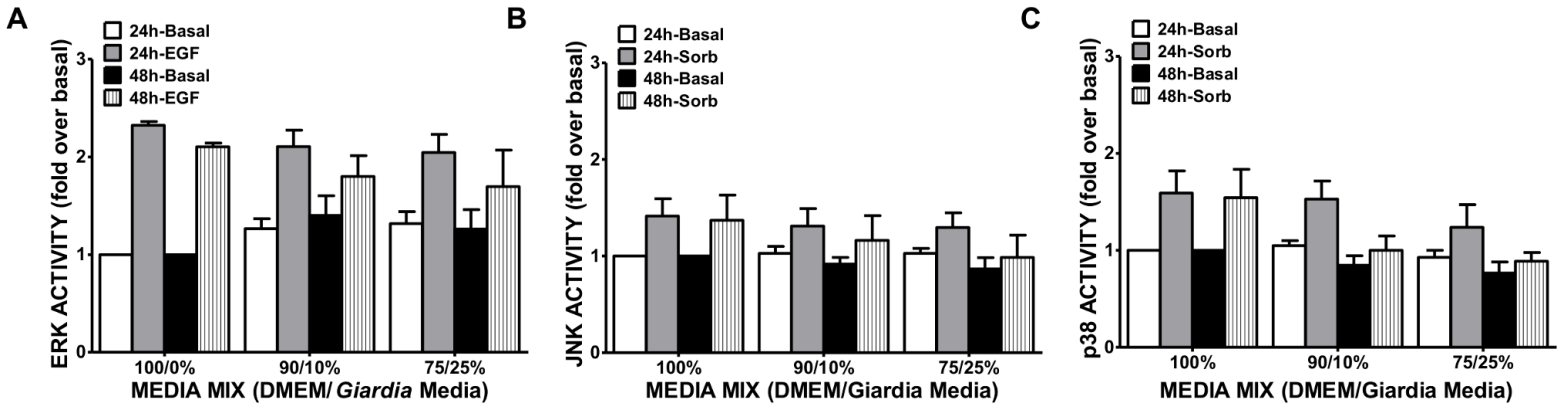
Effect of media mixes on MAPK activation in Caco-2 cells. Caco-2 cells were grown to confluence in 96-well plates, and cultured for 24 and 48 hours in the presence of the media mixes. Activation of stress-related kinases was measured using PACE assays. Activation of ERK (**A**), JNK (**B**), and p38 (**C**) was measured and compared to positive controls (EGF or sorbitol). Values are expressed as the mean fold over basal of 100% DMEM ± SEM. (n = 4).

Morphology of Caco-2 cells exposed to three media treatments, 100% DMEM, 90% DMEM/10% *Giardia* media, and 75% DMEM/25% *Giardia* media was assessed over 5 days. Caco-2 cells grown in 100% DMEM or the 90/10 mix maintain a defined shape and overall structured monolayer at 5 days. Caco-2 cell morphology showed the greatest deviation from control morphology in the 75% DMEM/25% *Giardia* medium treatment at 5 days (Figure 2A). In the 75/25 media mix, Caco-2 cells begin to exhibit an irregular shape at 2 days which becomes more pronounced by 5 days (Figure 2A). The monolayer appears to be somewhat disorganized indicating long-term culturing in this condition is not capable of maintaining the cellular characteristics of the epithelial cells. Caco-2 cell viability was assessed by trypan blue exclusion in the three media treatments. None of the media mixes had any effect on Caco-2 cell viability over the course of 7 days when compared to time-matched control cultures in 100% DMEM (Figure 2B). Basal activity of ERK, p38, and JNK in Caco-2 cells was compared in the three media treatments. Sorbitol, a known activator of p38 and JNK by inducing osmotic stress [42], and epidermal growth factor (EGF), which activates ERK signaling [43], were used as positive controls. Basal activity of the MAPKs did not differ between media treatments over 48 hours. While increasing *Giardia* medium concentrations caused modest decreases in Caco-2 cell responsiveness to positive stimuli (sorbitol or EGF) at 48 hours, these changes were not significant when compared to stimulation of control Caco-2 cells in 100% DMEM (Figure 3) (ERK, p = 0.5275) (JNK, p = 0.5695) (p38, p = 0.0920). Together, these results indicate that the 90% DMEM/10% *Giardia* media mix best supports Caco-2 cell growth based on cellular viability, morphology and MAPKs signaling; therefore, 90% DMEM/10% *Giardia* media combination was selected for future experiments.

### *Giardia* trophozoite proliferation and attachment in co-culture media

*G. lamblia* trophozoites preferentially colonize the small intestine where attachment to the epithelial barrier and proliferation within the gastrointestinal tract are essential for progression of infection in the host. *In vitro* studies have indicated that *Giardia* will not proliferate when cultured with Caco-2 cells grown in 100% DMEM [44], indicating these culture conditions do not accurately reflect *in vivo* conditions where parasites actively proliferate while attached to the epithelial surface. Attachment of trophozoites to epithelial cells *in vitro* is dependent on tonicity and pH of the growth medium [45]. In an attempt to better mimic a true *Giardia* infection, we evaluated parasite survival, proliferation and attachment in the co-culture media combination of 90% DMEM/10% *Giardia* media.

**Figure 4:**
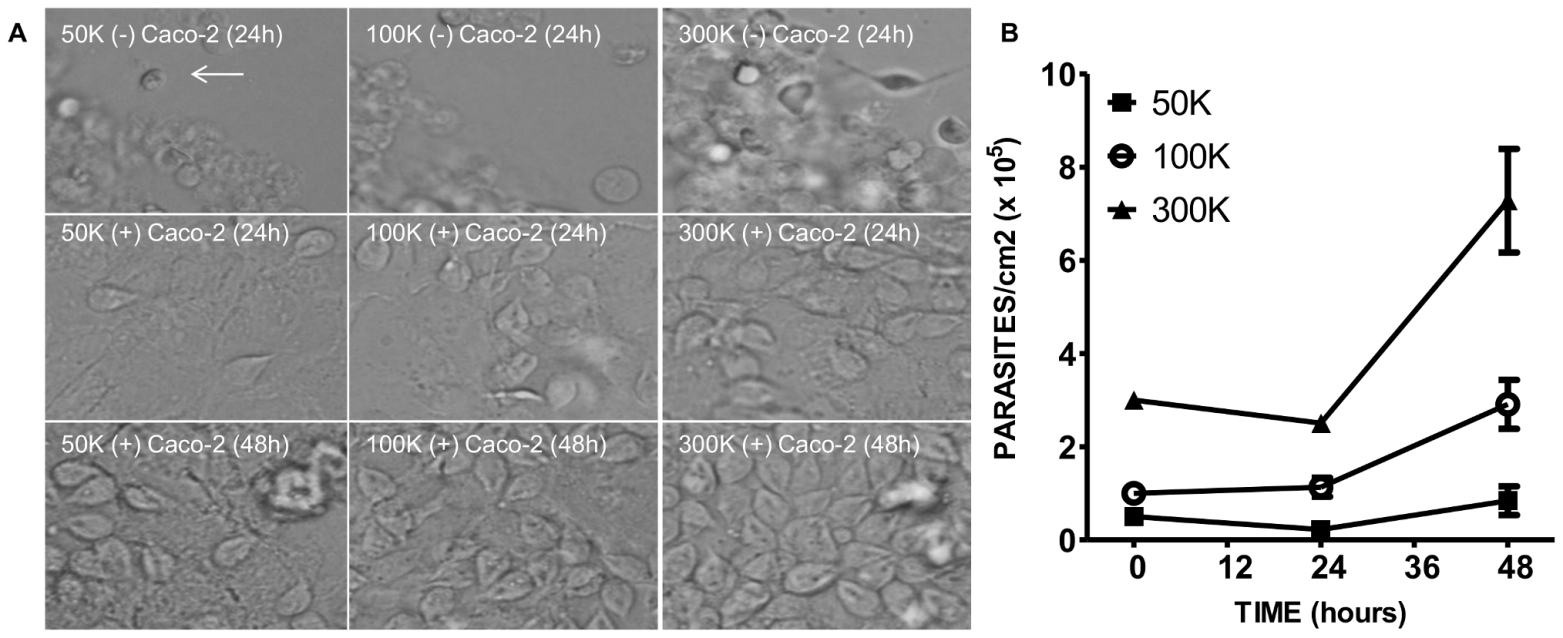
Proliferation of *Giardia* trophozoites in 90% DMEM/10%*Giardia* media at 3 starting densities. **A**) *Giardia* trophozoites were added to 24-well plates at three different starting densities: 50,000, 100,000, or 300,000 parasites/cm^2^ in the absence and presence of Caco-2 cells. **B**) Growth curve of *Giardia* co-cultured with Caco-2 cells over 48 hours at the three starting densities. Values are expressed as the mean ± SEM. (n = 3).

Using three starting densities of trophozoites, parasites were monitored for 48 hours in both the presence and absence of epithelial cells. In the absence of Caco-2 cells, trophozoites at all densities were not viable and did not proliferate over the course of 24 hours. Most of the trophozoites were found clumped together and had a rounded shape (Figure 4A, identified with an arrow). These spherical structures do not appear to be *Giardia* cysts as immunofluorescence microscopy analysis (IFA) using an antibody specific for the cysts failed to indicate cyst formation (supplemental data, Figure S1). Some of the round structures did label with the nuclear stain, DAPI; however, these were few in number and the staining was faint and diffuse. When co-cultured with Caco-2 cells in 90% DMEM/10% *Giardia* media, parasites displayed typical trophozoite morphology (Figure 4A) indicating the presence of epithelial cells increases parasite survival at low trophozoite density. Trophozoites upregulate many genes when cultured with epithelial cells, including those involved in oxygen defense, cell cycle regulation, and stage differentiation [46], [47], which could explain the survival of trophozoites in the presence of Caco-2 cells in this study. As previously suggested [44], 100% DMEM failed to support *Giardia* proliferation even in the presence of Caco-2 cells. IFA of trophozoites grown in the three media treatments for 24 hours show qualitatively fewer DAPI-positive parasites in the 100% DMEM treatment when compared to either the 90/10 or 75/25 mix (supplemental data, Figure S1). Additionally, the co-culture media combination of 90% DMEM/10% *Giardia* media allowed for parasite attachment to Caco-2 cells and proliferation over 48 hours (Figure 4B). Parasite attachment and proliferation are important parameters to the overall success of the parasite *in vivo*; therefore using the co-culture media combination in our model will allow certain aspects giardiasis to be recapitulated.

### Detachment of trophozoites using formononetin has no effect on MAPK signaling in Caco-2 cells

**Figure 5:**
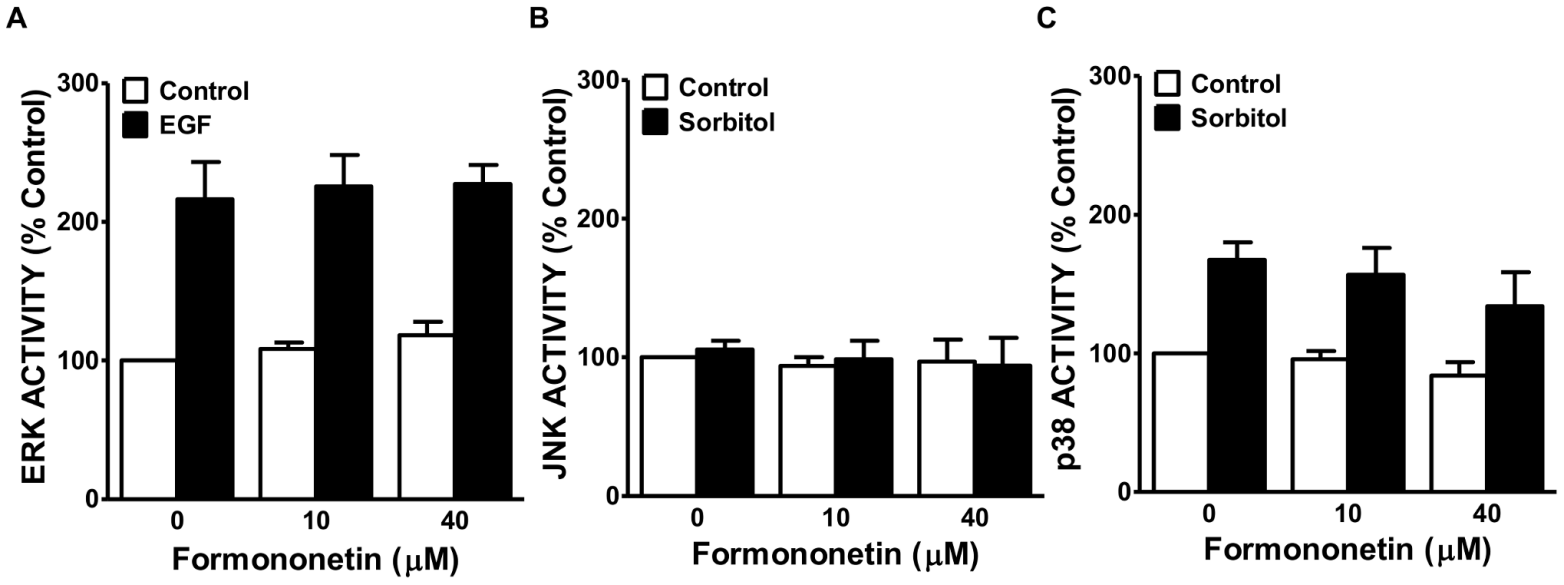
Effect of formononetin on stress activated kinases in Caco-2 cells. Caco-2 cells plated at 60,000 cells/ml were grown to confluence in 96-well plates. The cells were treated with control (DMSO) or 10 µM and 40 µM formononetin for 5 minutes. Activation of ERK (**A**), JNK (**B**), and p38 (**C**) were measured by PACE assays. EGF (100 ng/ml) treatment for 15 minutes was used as a positive control for ERK activation. Sorbitol (300 mM) treatment for 15 minutes was used to activate p38 and JNK. Values are expressed as the percent change over control of 100% DMEM ± SEM. (n = 4).

Traditionally, *Giardia* trophozoites are removed from surfaces, including epithelial cells, by cold shock [32]. However, cold shock treatment of cultured mammalian cells can induce physiological changes, including cytoskeleton rearrangement, p38 MAPK activation, and gene transcription activation [48]. Therefore, trophozoite removal by cold temperature exposure makes downstream studies of Caco-2 cells difficult to interpret. Preliminary work using our model demonstrated that using cold-shock washes to remove *Giardia* trophozoites damaged the Caco-2 monolayer due to the force employed to remove the parasites from the insert. As *Giardia* trophozoites can be chemically detached from surfaces using the isoflavone, formononetin [33], we assessed whether this compound could be used to remove trophozoites without affecting key apoptotic or proliferative signaling pathways in Caco-2 cells. Formononetin was added to confluent Caco-2 monolayers for 5 minutes as previously described [33] and then MAPK activities were assessed by PACE assay. Both 10 µM and 40 µM formononetin were able to detach *Giardia* trophozoites from Caco-2 cells [33] without altering MAPK activation in the Caco-2 cells (Figure 5). In addition, formononetin did not affect the response of Caco-2 cells to stimuli that activate ERK, JNK or p38 as similar levels of activation where observed in the presence of formononetin when compared to control stimulation with either sorbitol or EGF in the absence of the isoflavone compound (Figure 5). Trophozoites removed with this method were successfully re-cultured and were able to proliferate (data not shown). Although formononetin has anti-inflammatory properties [49] and can induce apoptosis [50], [51], increase NOS expression [52], and promote cell cycle arrest [53], none of these effects have been shown at the incubation time and/or concentration employed for *Giardia* removal. Based on these data, as well as the ability of both concentrations to efficiently detach more than 80% of parasites from cell layers within 5 minutes [33] without compromising the integrity of the Caco-2 cell monolayer, we used 10 µM formononetin treatments for 5 minutes to remove *Giardia* trophozoites.

### *Giardia* decreases epithelial cell number

**Figure 6:**
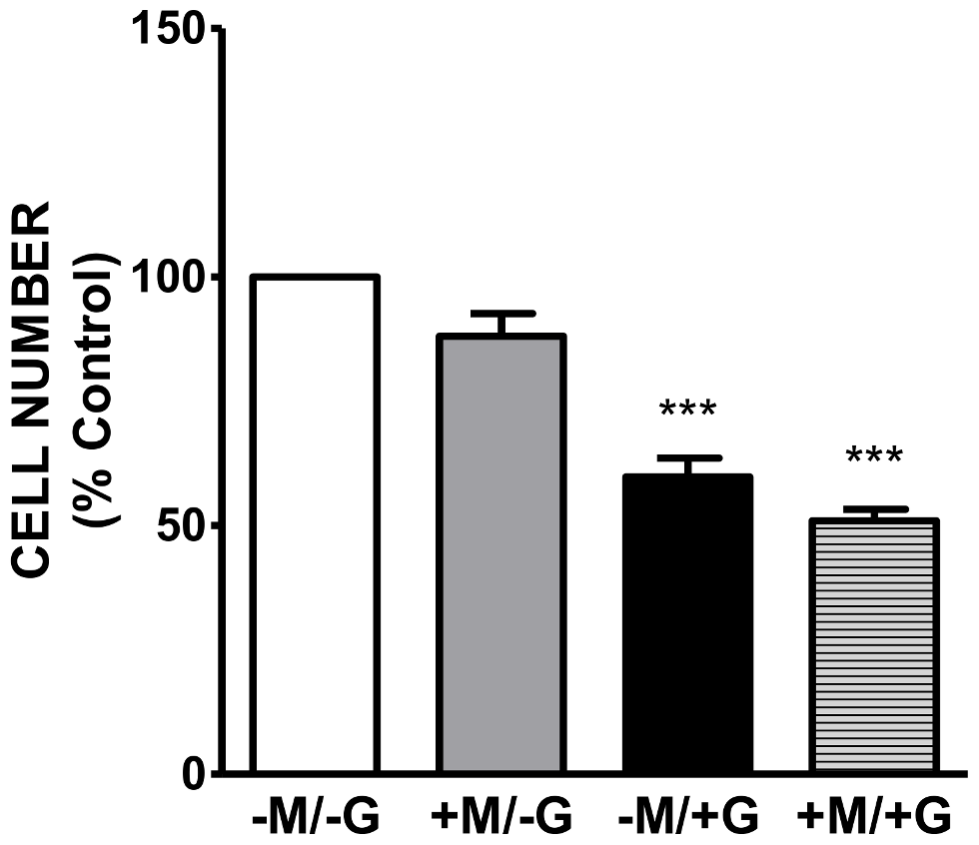
Effect of *Giardia* on Caco-2 cell number. *Giardia* trophozoites were added to the co-culture at 100,000 total parasites for 5 days in 90% DMEM/10% *Giardia* media. Methylene blue staining of Caco-2 cells alone, Caco-2 cells (+) macrophages, Caco-2 cells (+) *Giardia*, Caco-2 cells (+) macrophages (+) *Giardia*. Values are expressed as the% control of Caco-2 cells alone ± SEM. (p<0.0001) (n = 3).

Previous *in vitro* monolayer co-culture studies have shown decreased epithelial cell proliferation in response to *Giardia* interaction [15], [54]; however, co-culture studies with lung epithelial cells and activated macrophages have revealed that cytokine production by macrophages can induce epithelial cell proliferation as a mechanism to repair tissue damaged during the inflammation process [55]. To explore the role of macrophages during giardiasis, epithelial cell number was measured on cell culture inserts in the presence and absence of IC-21 macrophages during *Giardia* incubation. In the presence of *Giardia* trophozoites, there was a significant decrease in the number of epithelial cells at 5 days of incubation when compared to control (Figure 6). The presence of macrophages did not prevent the *Giardia*-induced decrease in Caco-2 cell number suggesting that macrophages do not increase epithelial cell proliferation during *Giardia* infections.

### *Giardia* increases caspase-3 activity in Caco-2 cells

**Figure 7:**
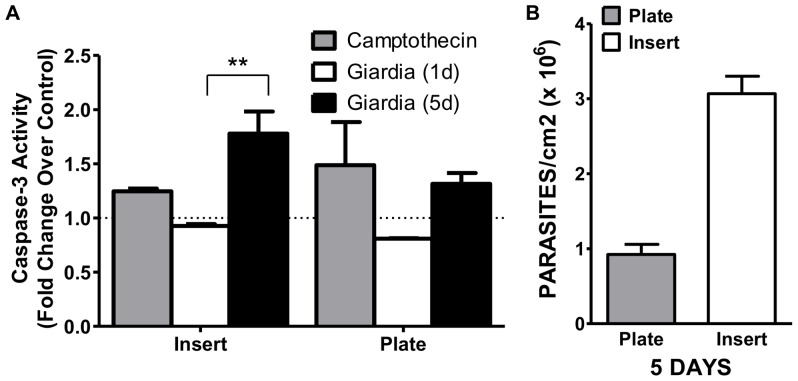
Caspase-3 activity in Caco-2 cells incubated with *Giardia*. **A**) Caco-2 cells grown in 6-well inserts or plates to confluence were inoculated with 350,000 parasites/cm^2^ *Giardia* trophozoites. Caspase-3 activation as a marker for apoptosis was measured at 1 and 5 days using the Abcam Caspase-3 assay kit. Camptothecin (5 µM) was used as an inducer of caspase-3 activation. The data represent the percent change over control ± SEM assayed three times in duplicate. (n = 3) (p = 0.0071) **B**) Parasite density at 5 days in the plate and insert culture conditions.

To investigate the mechanisms involved in epithelial cell number decrease during *Giardia* interaction, we measured the impact of *Giardia* on Caco-2 cell apoptosis. Caspase-3, activated by both the intrinsic and extrinsic pathways, is an executioner caspase that is responsible for both the morphological and biochemical changes associated with apoptosis (reviewed in [56]). Camptothecin, a strong inhibitor of DNA synthesis, is widely used to induce apoptosis *in vitro*. In our work, there was only a modest increase in caspase-3 activation in Caco-2 cells treated with camptothein. This result was somewhat expected as Caco-2 cells are less responsive to camptothecin after confluence and throughout the differentiation phase [57]. The activity of caspase-3 in Caco-2 cells was assessed at 1 and 5 days in both plate and insert conditions. Overall, the basal level of caspase-3 activity is higher in the insert when compared to the plate culture; however, unstimulated caspase-3 levels in insert control cultures do not vary from 1 to 5 days. The difference in baseline caspase-3 activity between the two experimental conditions may reflect different Caco-2 phenotypes that exist in the two culture parameters [58]. At 1 day there is no change in caspase-3 activity in either plate or insert cultures when Caco-2 cells are incubated with *Giardia* (Figure 7A); however, at 5 days there is a significant increase in caspase-3 activity in the insert cultures, but not in the plate culture (Figure 7A). Additionally, even though the plate and insert cultures were inoculated with the same starting density of parasites, at the termination of 5 days, parasite density is much higher in the insert when compared to the plate environment (Figure 7B). This suggests the insert environment better supports the growth requirements of *Giardia* trophozoites.

### Modulation of host cytokines by *Giardia*

**Figure 8:**
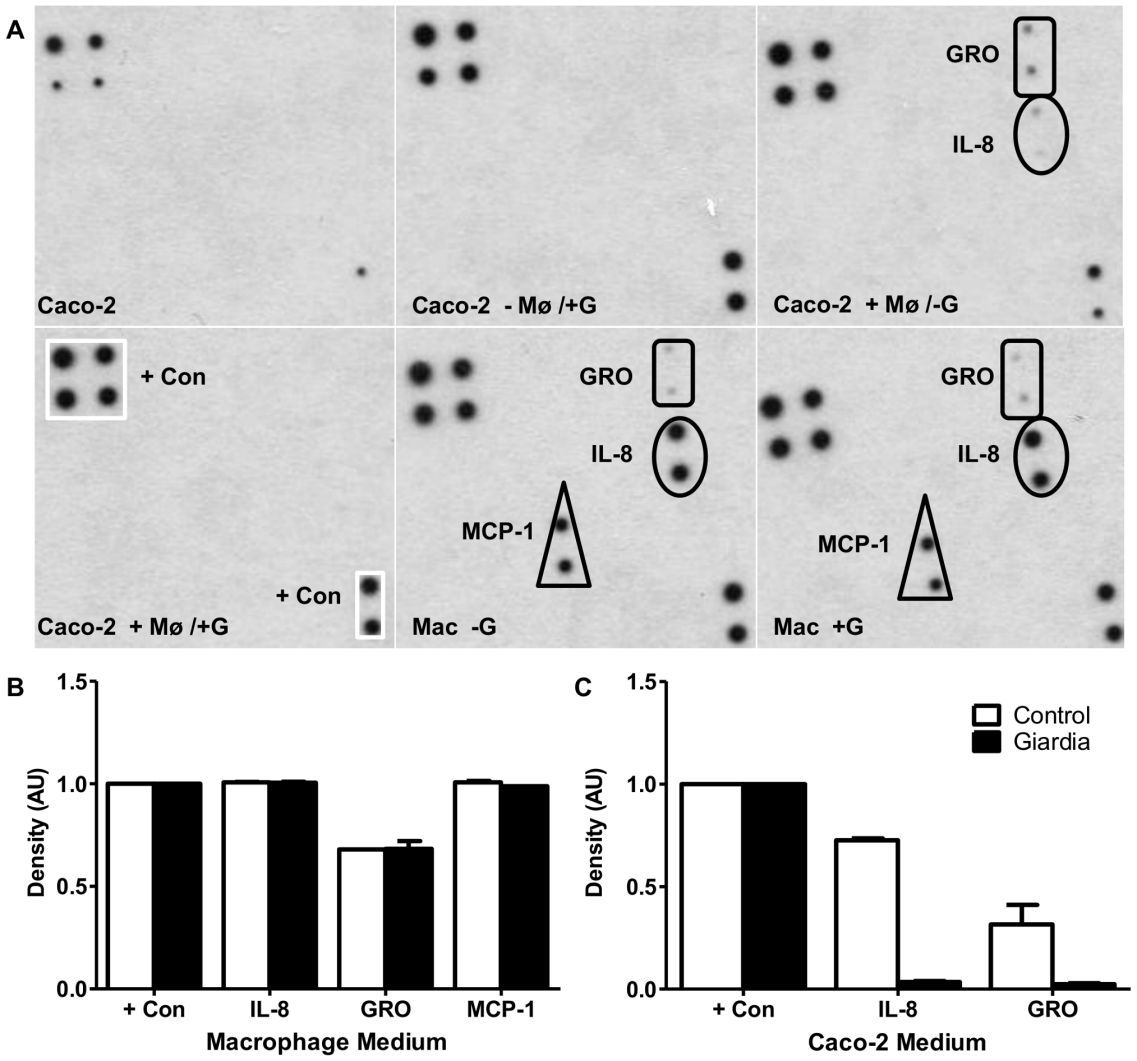
Cytokine profile of Caco-2 cells and macrophages in the co-culture system. Caco-2 cells were cultured in the presence and absence of human differentiated macrophages for 3 days. *Giardia* trophozoites were added and incubated for 5 days. **A**) Conditioned medium was collected and analyzed using the RayBiotech cytokine array (supplemental data, Figure S2). **B**) Macrophage medium blot intensity as determined by ImageJ. **C**) Caco-2 medium blot intensity as determined by ImageJ.

Cytokines are important in determining the type of adaptive immune response initiated while also advancing the innate response [59]; however, little is known about these processes in giardiasis. Interleukin-6 (IL-6) [60] and TNF-α [61], [62], [25] have been implicated in the host immune response against *Giardia*. Roxstrom-Lindquist et al. [15] found increased expression of cytokines involved in recruitment of immune cells from *Giardia-* exposed Caco-2 human intestinal cells. These findings indicate that immune cells may contribute to modulating *Giardia* infections. Using cytokine arrays, the cytokine profile of macrophages and Caco-2 cells were characterized following 5 days of co-culture in the absence and presence of *Giardia*. Each array contains capture antibodies printed on a nitrocellulose membrane that detects both the presence and relative expression levels of 23 human cytokines (Supplemental data, Figure S2). Caco-2 cells grown on inserts alone failed to secrete any of the cytokines tested (Figure 8); however, when grown in the presence of differentiated macrophages, Caco-2 cells secreted GRO and IL-8 (Figure 8), which both act in chemoattraction and neutrophil activation (reviewed in [63]). Incubation of the Caco-2 cell-macrophage co-culture with *Giardia* abolished the secretion of both GRO and IL-8 cytokines from Caco-2 cells (Figure 8). Differentiated macrophages secreted GRO, MCP-1, and IL-8 when co-cultured with Caco-2 cells and this response was not altered by *Giardia* (Figure 8).

### Long-term viability of cells and parasites in the co-culture model

**Figure 9:**
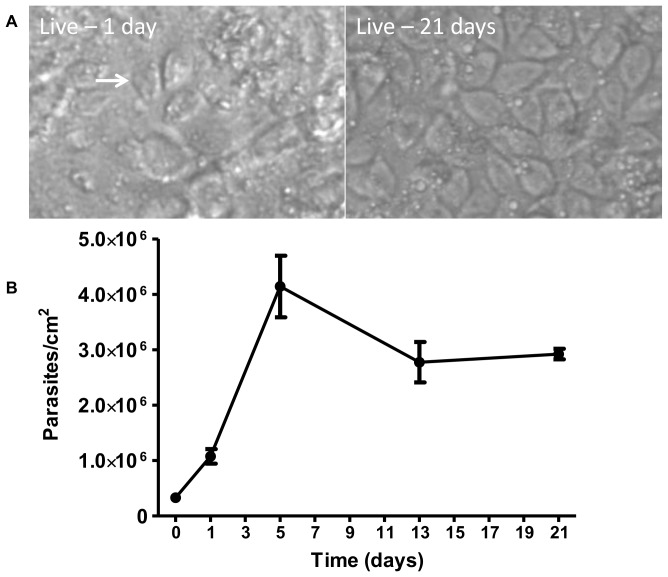
*Giardia* trophozoites in the 21-day co-culture. The co-culture model was assembled as described. **A**) Live images of the model with parasites attached were obtained at 1 and 21 days. A trophozoite is identified with an arrow. Full size images of the model at day 1, 5, 13, and 21 are provided in the supplemental data (Figures S3–S6). **B**) *Giardia* growth curve over 21 days in the co-culture model. Trophozoites removed from the inserts with formononetin treatment were collected and counted with a hemocytometer at 1, 5, 13, and 21 days. Data represents the mean of four individual insert counts ± SD.

**Figure 10:**
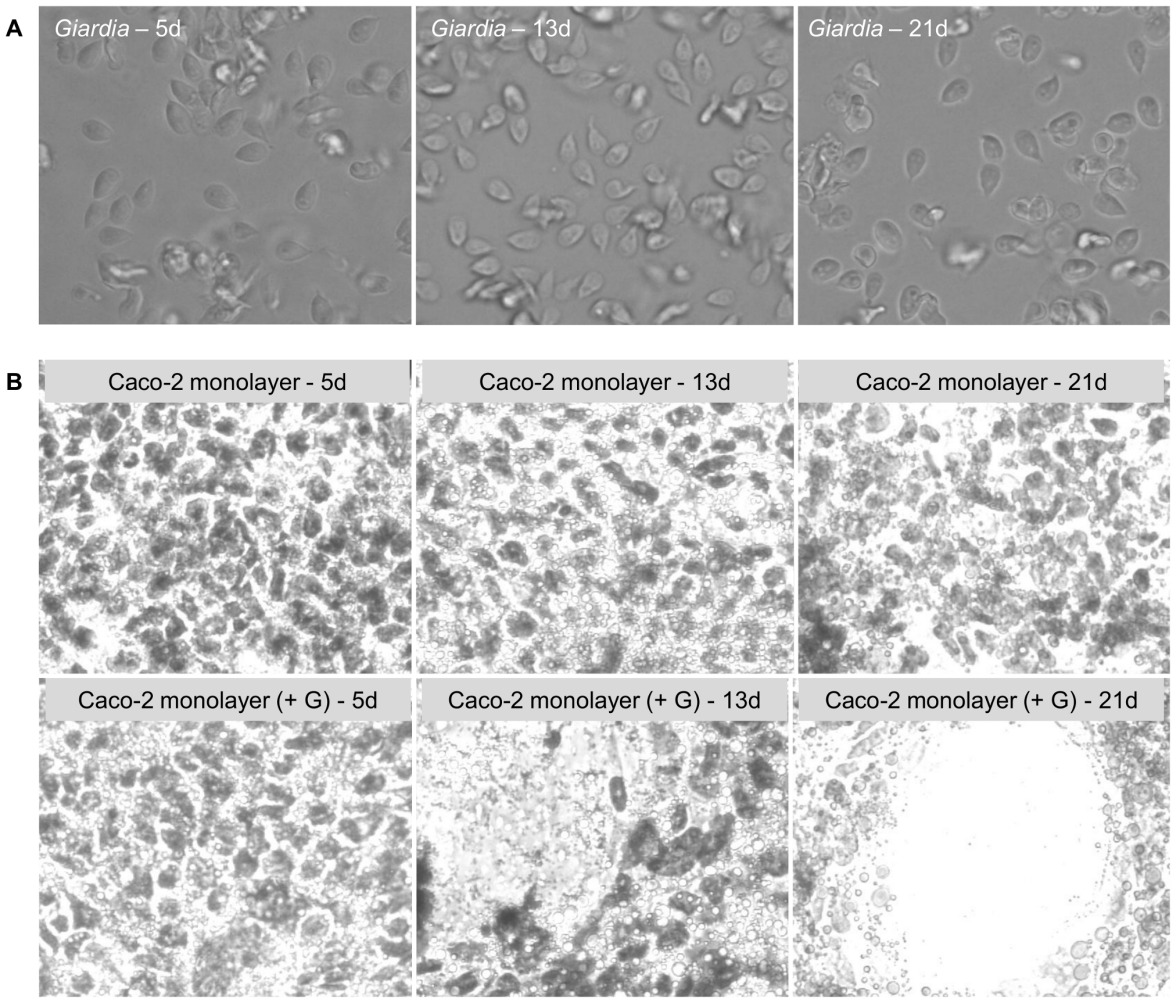
Morphology of *Giardia* and Caco-2 cells over 21 days. **A**) Images of trophozoites removed from co-culture inserts with formononetin treatment at 5, 13, and 21 days. **B**) Crystal violet stain of the Caco-2 monolayer on inserts in the absence or presence of *Giardia* at 5, 13 and 21 days.

Since *Giardia* infections can span several days to weeks in both mice [64] and humans [65], [14], constructing a model that allows for long-term incubation of parasites with epithelial cells is essential to understanding long-term infection interactions on the cellular level. Using the co-culture model, parasite viability and proliferation, as well as Caco-2 cell monolayer structure was monitored over 21 days. Parasites remained viable in the co-culture model for the full 21 days (Figure 9A) and completely covered the Caco-2 monolayer by day 5 (supplemental data, Figures S3–S6). Parasite density peaked at 5 days and then reached a steady state density that persisted to 21 days (Figure 9B). The decline in parasite number after day 5 is attributed to the increased frequency of feeding after that time. Trophozoites removed from the co-culture retained typical trophozoite morphology (Figure 10A). The Caco-2 monolayer remains attached to the insert, but had started to disorganize by 13–21 days as indicated by the presence of holes in the monolayer (Figure 10B). Disruption of the Caco-2 monolayer is *Giardia* mediated as control inserts containing no parasites showed no structural damage over the 21 day time period (Figure 10B). Additionally, macrophages can be observed on the bottom of the insert (supplemental data, Figure S7); however, by 21 days few macrophages remained on the insert and were mostly found in the bottom of the well. These data indicate that our model allows for long-term survival of *Giardia* and epithelial cells *in vitro* as the Caco-2 cell monolayer is preserved in control culture conditions and trophozoite survival, morphology, and attachment is conserved over the span of 21 days.

## Discussion

Several co-culture models of the human intestine have been developed [37], [66]; however, no experimental design has been adapted for prolonged *Giardia*-host interactions. The use of transwell inserts for *Giardia* research has been established for short-term studies of 24 hours or less [15], [67]. Long-term culturing of *Giardia* trophozoites with human epithelial cells *in vitro* is challenging [46], [6] due to the microaerophilic nature of the parasite. To overcome the parasite survival barrier, most *in vitro* studies have utilized high multiplicities of infection in addition to the short incubation times. These conditions do not reflect a typical *Giardia* infection where a low infectious dose leads to an active infection that can span several days to weeks. Therefore, establishing an *in vitro* model that allows for the protracted co-culture of host epithelial and immune cells with *Giardia* trophozoites would greatly contribute to the understanding of late infection interactions.

Our model utilizes transwell inserts to co-culture a human intestinal epithelial cell line (Caco-2) and a murine peritoneal macrophage cell line (IC-21) in a manner that represents the apical-basolateral orientation of the small intestine (Figure 1). IC-21 murine peritoneal macrophages were selected for their similarity to human macrophages, including their typical macrophage morphology in culture, their expression of macrophage-specific antigens, their activation by lipopolysaccharide, their phagocytic ability [31], and their expression of IgG receptors [68]. Caco-2 cells, derived from a human colonic adenocarcinoma retain both morphologic and phenotypic characteristics of intestinal epithelial cells when fully differentiated, including polarized morphology, microvilli on the apical surface, expression of brush border enzymes, and adjacent cell tight junctions (reviewed in [69], [58]). The *in vitro* differentiation of Caco-2 cells into a phenotype similar to small intestinal epithelial cells is a time-mediated event that is dependent on many factors, including passage number, seeding density, media composition, and substrate support (reviewed in [58]). In our study, the use of Caco-2 cells at three days post plating allows for the assessment of how the parasite affects the proliferation and differentiation process of enterocytes in the intestine. The epithelial barrier of the intestine is replenished every 4–5 days; therefore, enterocyte renewal through stem cell differentiation is critical for normal functioning of the human gut (reviewed in [70], [71]). Caco-2 cells have been used to model the differentiation process of enterocytes in the small intestine [72]. Although immature proliferating Caco-2 cells show differences in gene expression [73], the protein expression profiles are remarkably similar [74] when compared to fully differentiated Caco-2 cells. Thus far, Caco-2 cells are the best described enterocyte cell line and the most common epithelial cell line used in *in vitro Giardia*–host interactions. Therefore, using Caco-2 cells to characterize our co-culture model allows us to compare our results with those published in the literature.

Following establishment of cell-cell communication and epithelial monolayer formation, *Giardia* trophozoites were added to the system using a lower starting density than what has previously been reported. This model allows the parasite to proliferate in culture, more accurately reflects the infection *in vivo*, and allows for the characterization of host-*Giardia* interactions from the start of an infection through its termination, including the role (if any) of immune cells in limiting the infection. Our data indicate that both parasites and epithelial cells are viable in the 90% DMEM/10% *Giardia* media mixture. Using transwell inserts filled with medium, we were able to limit the oxygen exposure of the parasites, while allowing the epithelial cells to exchange oxygen and nutrients through their basolateral surface. *Giardia* proliferates in the 90% DMEM/10% *Giardia* media and saturates the insert surface at 5 days post-infection. The drop in parasite density after 5 days is attributed to daily feeding of the insert cultures. Increased metabolism in co-cultures modeling infectious disease can drastically alter the media composition, pH, and by-product accumulation in the system [28]; therefore, a feeding strategy must be employed to offset these effects. We elected to only remove half the culture medium every other day until day five so that the host-parasite interface remained undisturbed until the infection was established. After five days, parasite density peaked and unattached parasites had to be removed daily. This approach mimics *in vivo* conditions where unattached parasites are pushed down the intestine and are ultimately excreted from the host. Although the 90/10 media mix does not support trophozoite survival in the absence of Caco-2 cells in plate cultures, we have shown inserts provide a more suitable environment for trophozoite proliferation. Therefore, further optimization of the insert model, such as altering parasite inoculation density or media composition, could allow parasite survival in the absence of host cells. This would make assessment of host-induced changes in *Giardia* gene expression feasible using our developed model. Our experimental design recapitulates the architecture of the gastrointestinal tract where *Giardia* trophozoites attach apically to the epithelium and epithelial cells interact basolaterally with macrophages in the lamina propria. Using human differentiated macrophages isolated from buffy coats for the cytokine array further illustrates the versatility of the model. However, due to low isolation numbers, lack of proliferation, and difficulty in maintaining the human monocyte-derived macrophages in culture, IC-21 cells were used in all other experiments to characterize our model.

Epithelial cell apoptosis as a mechanism of barrier dysfunction during giardiasis has been well documented *in vitro* [19], [18], in human biopsies [17], and in mouse models with *G. muris* [75]. However, the results of those experiments have been contradictory with regards to the degree of apoptosis observed as well as the *Giardia* assemblage(s) capable of inducing epithelial cell death. Studies using sonicated *Giardia lamblia* strain WBC6 (assemblage A), failed to elicit epithelial cell apoptosis [19]; a finding inconsistent with other work using live WBC6 trophozoites [18]. To assess apoptosis in our model, we compared caspase-3 activation in Caco-2 cells on inserts to the long-established monoculture plate environment. Our results indicate that live WBC6 trophozoites can induce apoptosis in a time-dependent manner. Since sonicated *Giardia* WBC6 parasites fail to produce the same response [19], this may indicate this particular *Giardia* strain mediates host cell death through a direct parasite-epithelial cell interaction. The difference in apoptosis observed between plate and insert cultures at 5 days in our studies is likely due to parasite density in the different culture conditions. Significantly more parasites are observed in the insert environment even though the plate and insert cultures received the same starting density of parasites. We speculate that the reduced parasite proliferation in the plate is due to high oxygen tension, which is deleterious to *Giardia* trophozoites [76]. Together, these data suggest that our model is a better representation of giardiasis as parasites can reach higher densities in the insert environment and remain viable over many days. Indeed, the contradictory data on apoptosis during giardiasis is likely due to many factors including *Giardia* strain utilized, parasite density, and type of epithelial cell line used.

The cytokine array illustrated the importance of co-culture in modulating cell phenotype. Caco-2 cells exhibit a different cytokine profile in the presence of human differentiated macrophages, which has been previously reported [77]. Secretion of chemotatic cytokines, such as GRO isoforms and MCP-1, by intestinal epithelial cells incubated with *Giardia* has been reported [15]. However, *Giardia* failed to elicit secretion of these cytokines from Caco-2 cells cultured alone and, in fact, suppressed the cytokine expression of Caco-2 cells cultured with macrophages by abolishing IL-8 and GRO secretion. Immuno-regulation of host defenses has been observed in *Giardia* [78] and other parasitic infections (Reviewed in [79], [59]). Differences in experimental design and Caco-2 cell differentiation state between the two studies as well as parasite density could explain some of the disparities. Furthermore, differences in the cytokine profile of intestinal epithelial cells during *Giardia* infection could be attributed to *Giardia* assemblage. *In vitro*, *Giardia* strain WB does not induce IL-8 secretion (shown here and [15]) while *Giardia* strain GS, belonging to assemblage B, elicits a robust IL-8 response in epithelial cells [80]. Differences in virulence between assemblage A and B have been previously suggested [10]–[12].

The role of macrophages in human giardiasis has yet to be fully resolved. Macrophages can actively phagocytose trophozoites *in vitro* [23] and in infected mice [21]. However, only low numbers of macrophages are observed in the lumen of *Giardia* infected animals [81]; therefore, phagocytosis alone probably does not contribute greatly to parasite control as *Giardia* is mostly non-invasive and does not cross the epithelial barrier. Using the co-culture model, we assessed if macrophages play a role in *Giardia* pathology by secreting cytokines or stimulating epithelial cell proliferation. In other tissues, macrophage cytokine secretion can activate epithelial cell proliferation as a means to repair damage or maintain homeostasis [82], [55]. In our studies, macrophages did not induce proliferation of Caco-2 cells exposed to *Giardia* parasites. The decrease in Caco-2 cell number in the presence of *Giardia* is likely due to epithelial cell apoptosis. The increase in caspase-3 activity in Caco-2 cells incubated with *Giardia* was quite substantial as it surpassed the levels induced by the camptothecin, a strong inhibitor of DNA synthesis. The dissociation between macrophages and epithelial cell proliferation in the gastrointestinal tract could be in part due to the unresponsive, anergic nature of intestinal macrophages (reviewed in [83], [84]). Additionally, macrophages do not control *Giardia* infection through cytokine secretion as the cytokine profile of the macrophages did not change in the presence of parasites. If this is due to regulation of epithelial cell cytokine secretion by the parasite is yet to be determined.

In summary, we have developed a model that allows for the long-term characterization of host*-Giardia* interactions, including the role immune cells play in parasite control and/or clearance. As monolayer integrity is compromised at later time points, this model can investigate disease physiology, such as altered transport function leading to malabsorption, until about 13 days post-infection. Incubations spanning longer than 13 days can be utilized for pathological studies. This model can be adapted to define culture conditions for the long-term culture of other *Giardia* strains, which will allow for the identification of strain-specific effects on host cells that may contribute to the wide spectrum of disease symptoms and infection duration. In addition, using the co-culture model for additional characterization of cytokine profiles unique to *Giardia* infections will provide insights into the underlying mechanisms of host immune suppression by the parasite. Overall, this model can help identity mechanisms of disease in giardiasis that can then be used as targets of therapeutic intervention.

## Supporting Information

Figure S1 **Immunofluorescence microscopy analysis of parasites in the media mixes.** *Giardia* trophozoites (50,000 parasites/cm^2^) where incubated in the three media mixes for 24 hours both in the presence and absence of Caco-2 cells. Parasites were incubated with a cyst specific antibody (green). Nuclei were stained with DAPI (blue). Pictures represent the merged images of DAPI and cyst antibody.
(TIF)

Figure S2 **RayBio® Human Cytokine Antibody Array map.**
(TIF)

Figure S3 **Live image of co-culture model at 1 day.**
(TIF)

Figure S4 **Live image of co-culture model at 5 days.**
(TIF)

Figure S5 **Live image of co-culture model at 13 days.**
(TIF)

Figure S6 **Live image of co-culture model at 21 days.**
(TIF)

Figure S7 **IC-21 macrophages in the co-culture.** IC-21 macrophages on the bottom of the insert were fixed in 4% formaldehyde and stained with 4% crystal violet. A macrophage is identified with an arrow.
(TIF)

Video S1 **Video of co-culture at day 0.** The co-culture consisting of Caco-2 cells and IC-21 macrophages was assembled as described and incubated for 3 days. *Giardia* trophozoites were then added at 100,000 total parasites and a video was taken immediately.
(AVI)
