# Supplementary figures and images for: Modeling Long-Term Host Cell-Giardia lamblia Interactions in an In Vitro Co-Culture System
Source: PLoS One. 2013 Dec 3;8(12):e81104. doi: 10.1371/journal.pone.0081104 (PMC3849038; doi:10.1371/journal.pone.0081104)

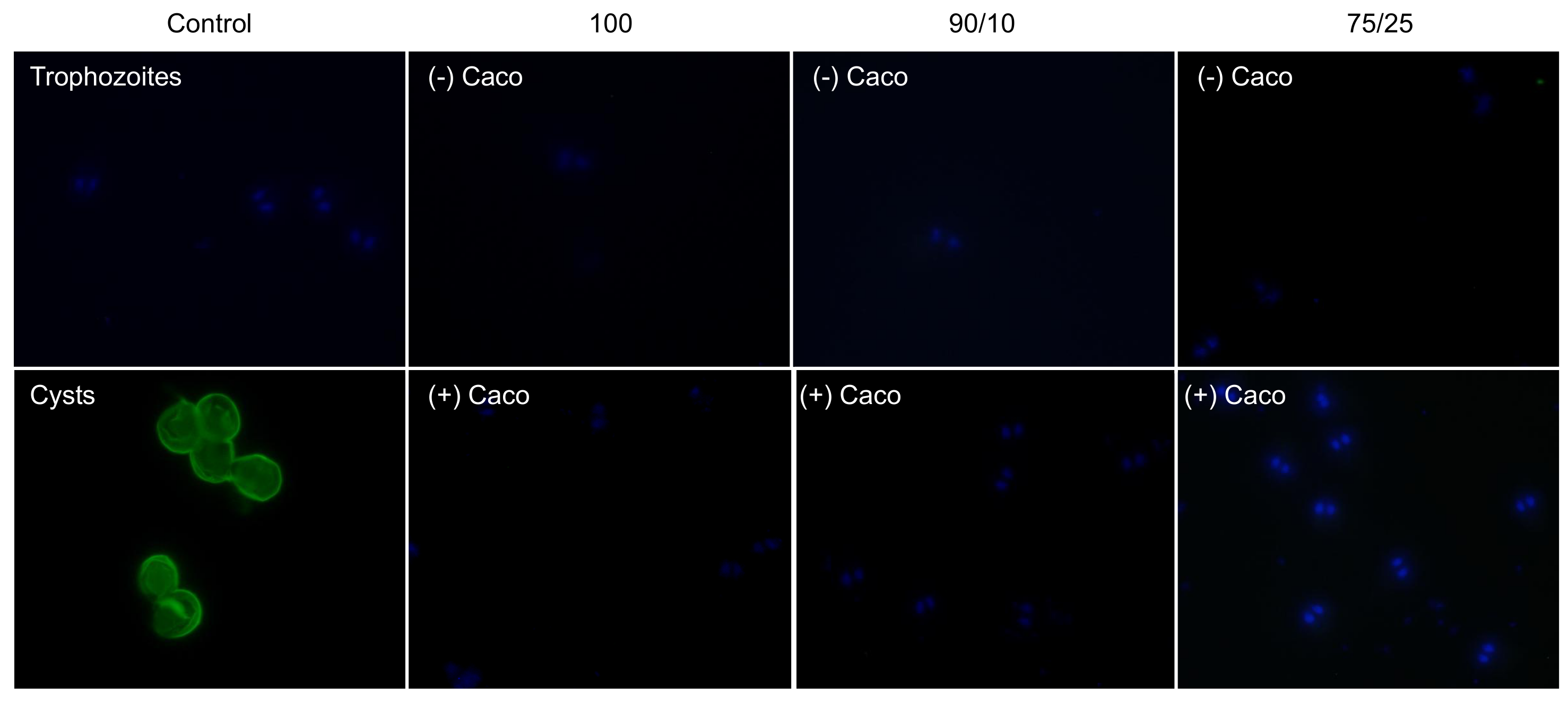

Supplement: Figure S1 — Immunofluorescence microscopy analysis of parasites in the media mixes. Giardia trophozoites (50,000 parasites/cm2) where incubated in the three media mixes for 24 hours both in the presence and absence of Caco-2 cells. Parasites were incubated with a cyst specific antibody (green). Nuclei were stained with DAPI (blue). Pictures represent the merged images of DAPI and cyst antibody. (TIF) [file pone.0081104.s001.tif]

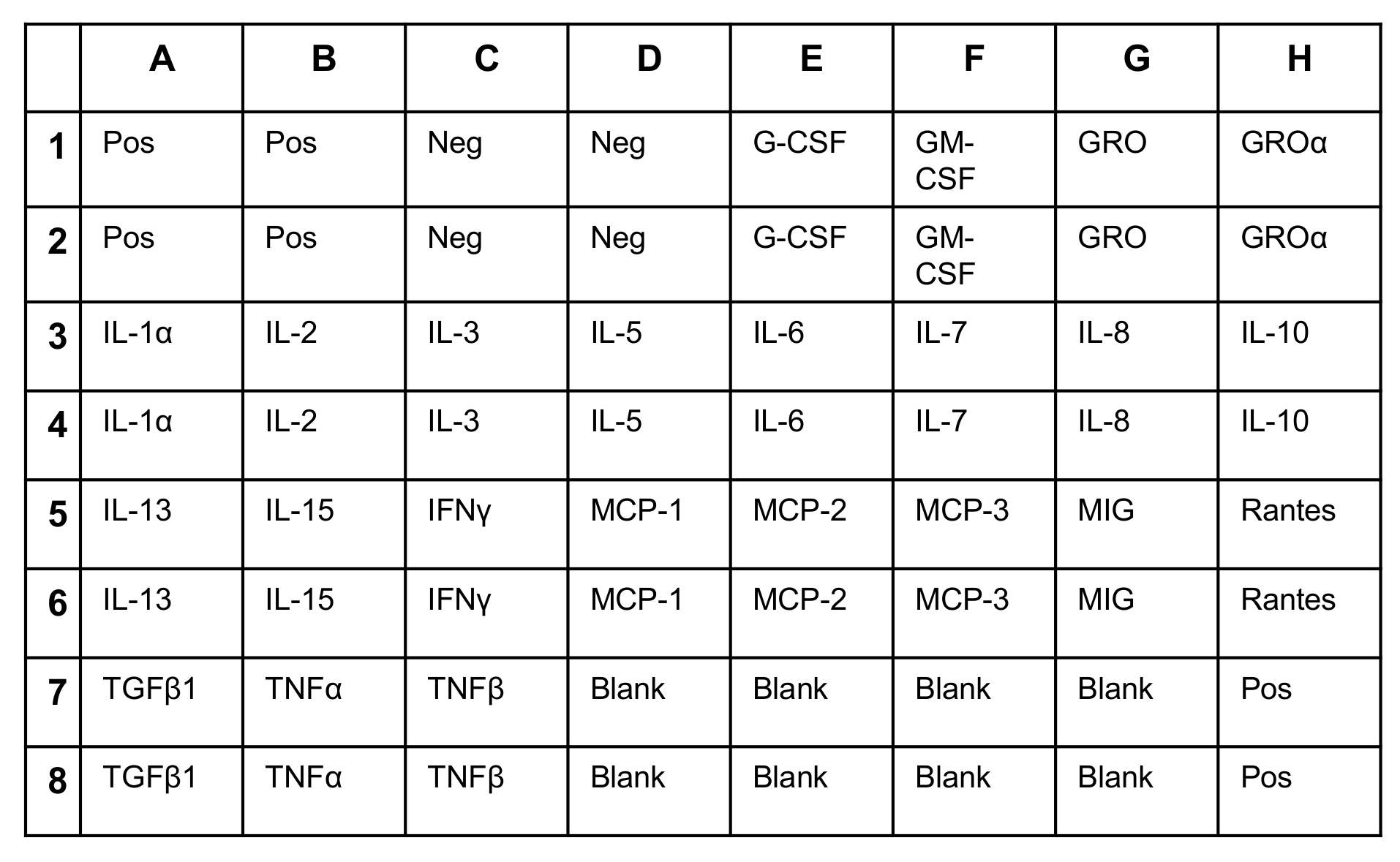

Supplement: Figure S2 — RayBio® Human Cytokine Antibody Array map. (TIF) [file pone.0081104.s002.tif]

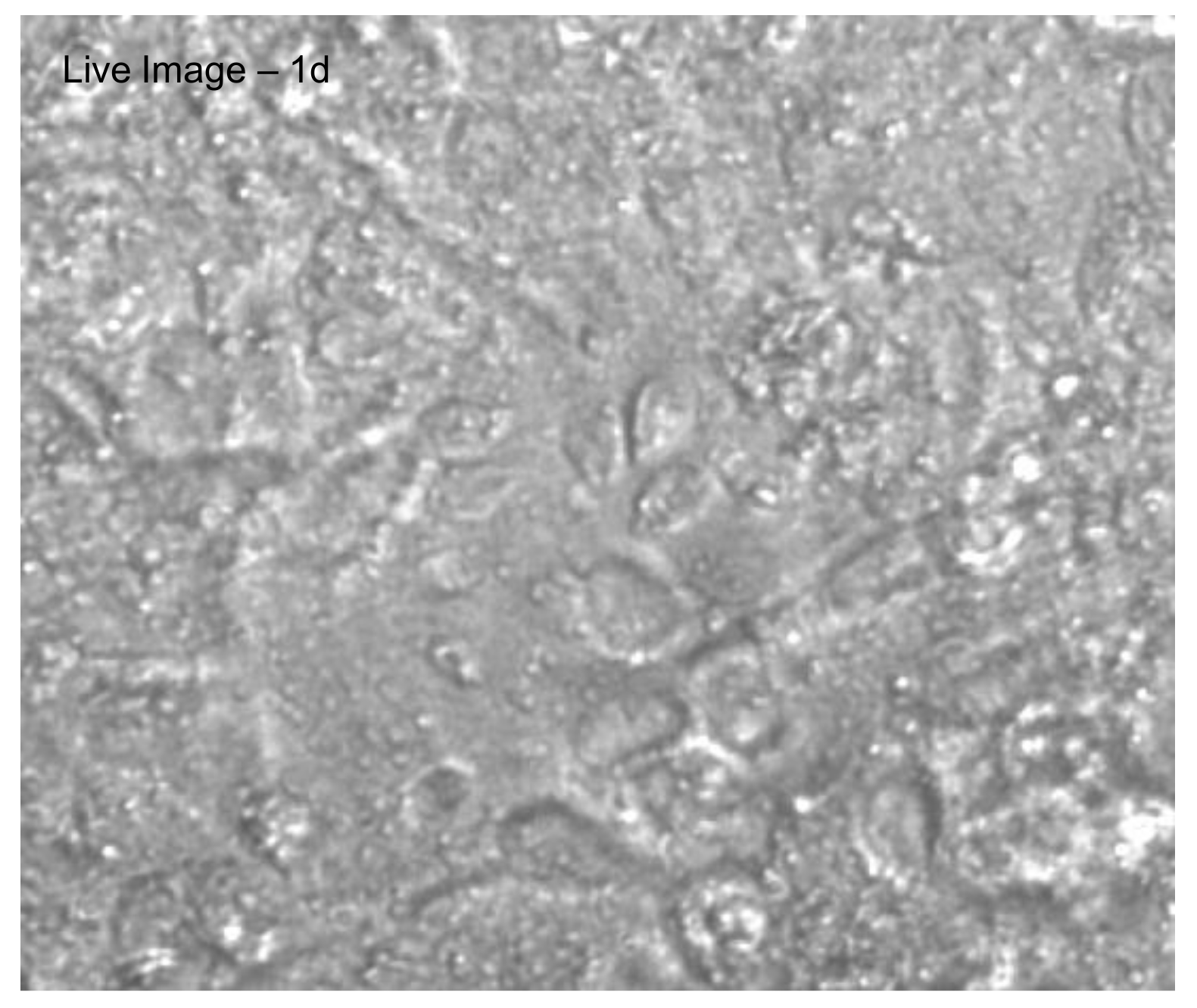

Supplement: Figure S3 — Live image of co-culture model at 1 day. (TIF) [file pone.0081104.s003.tif]

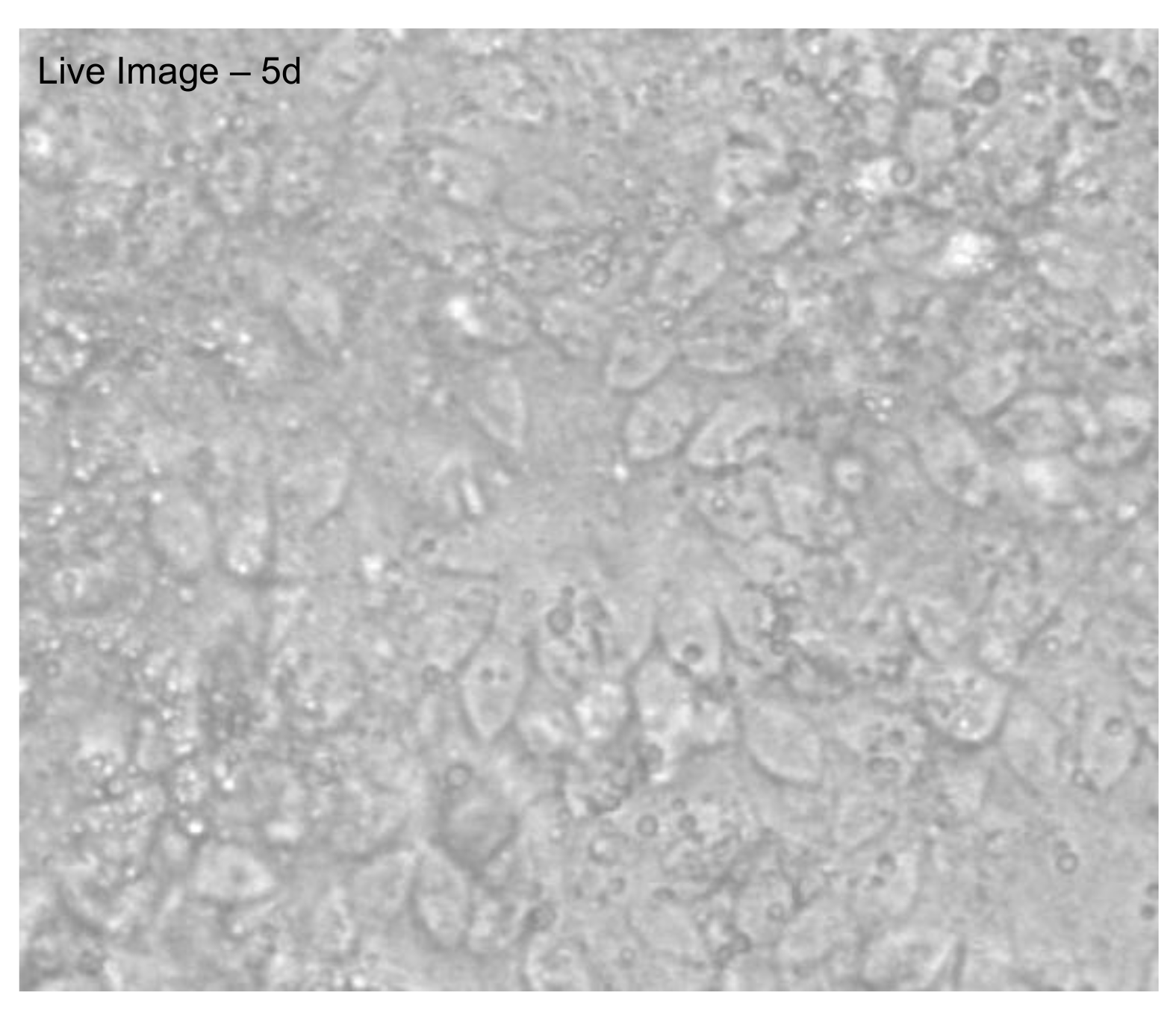

Supplement: Figure S4 — Live image of co-culture model at 5 days. (TIF) [file pone.0081104.s004.tif]

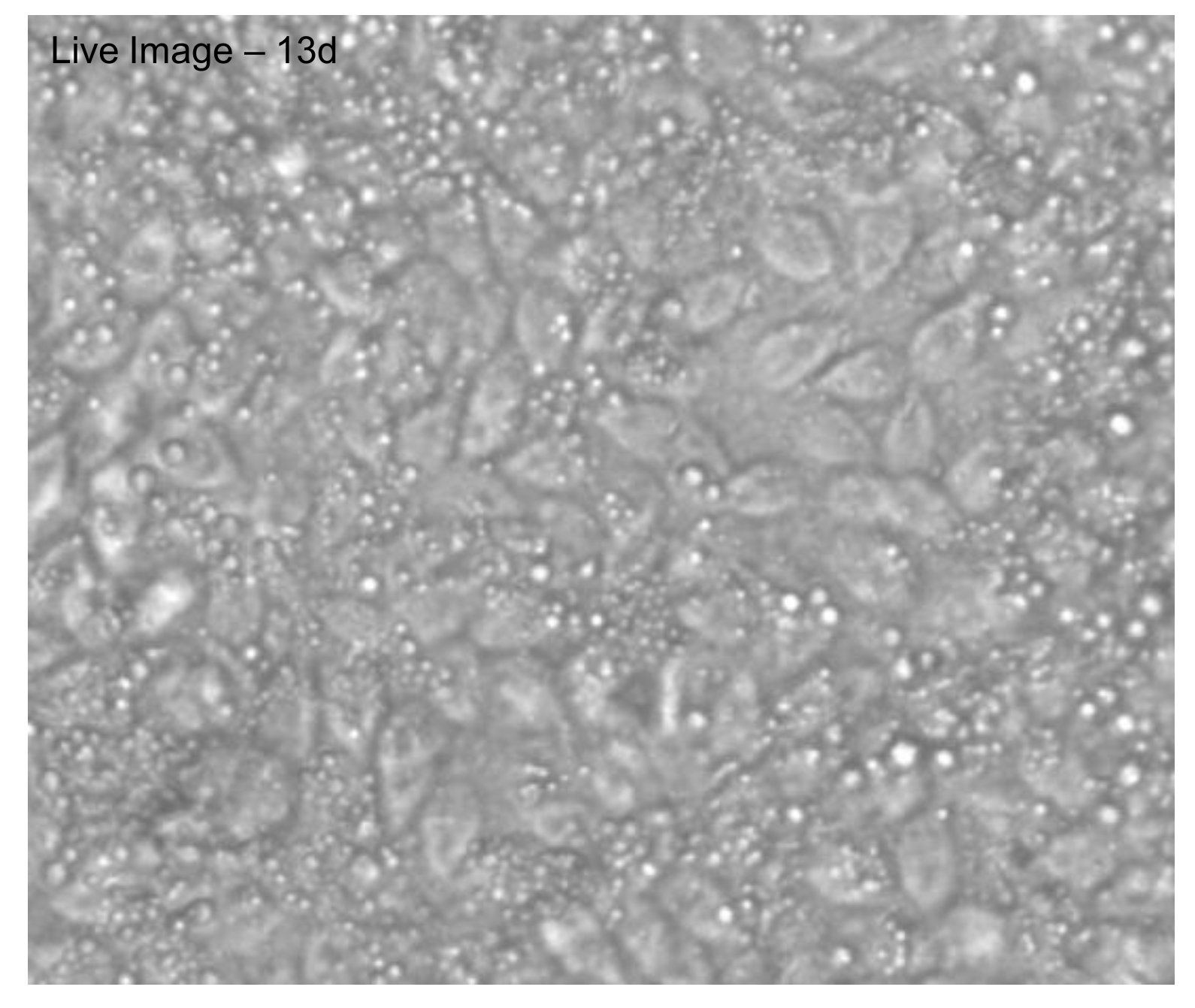

Supplement: Figure S5 — Live image of co-culture model at 13 days. (TIF) [file pone.0081104.s005.tif]

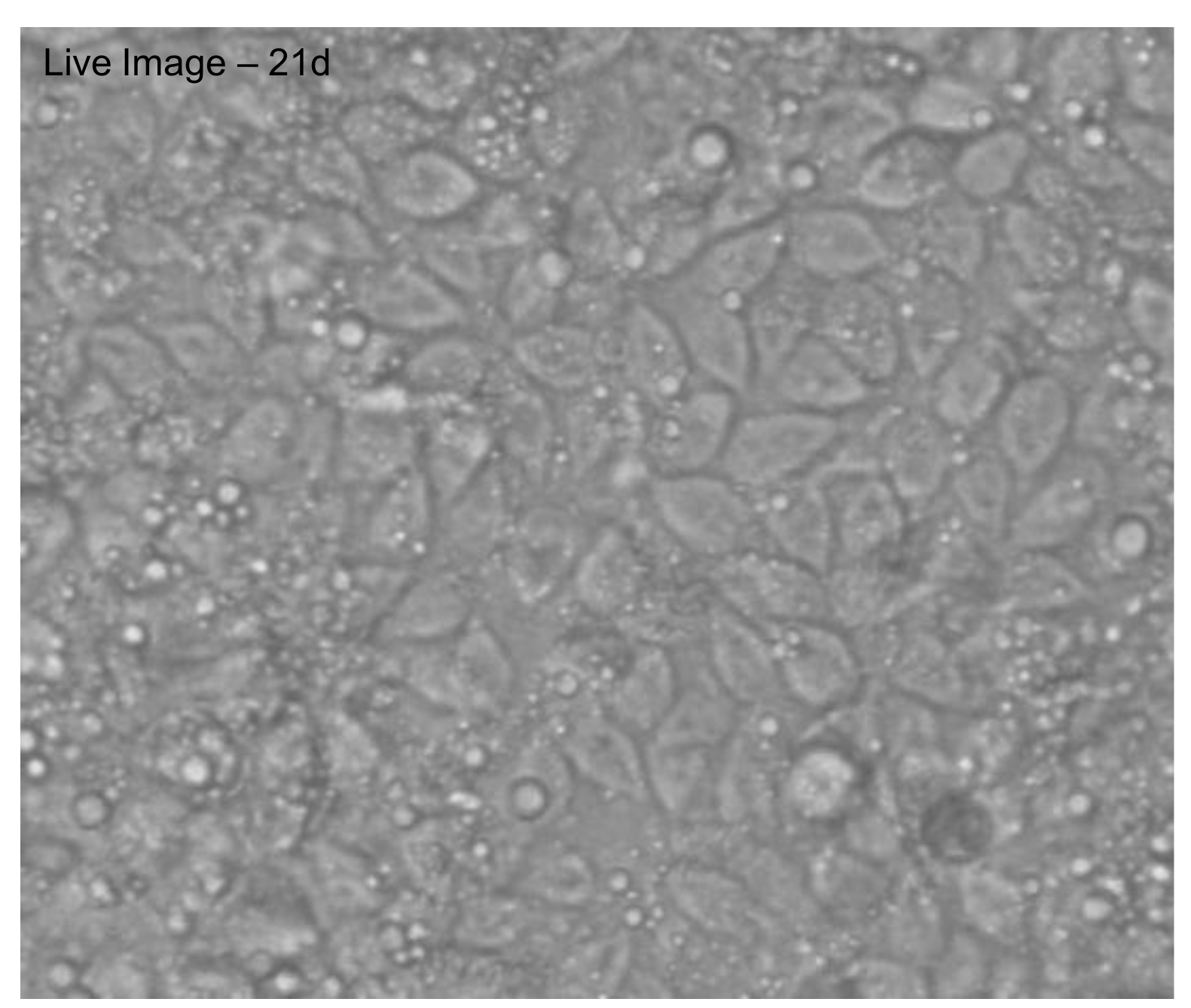

Supplement: Figure S6 — Live image of co-culture model at 21 days. (TIF) [file pone.0081104.s006.tif]

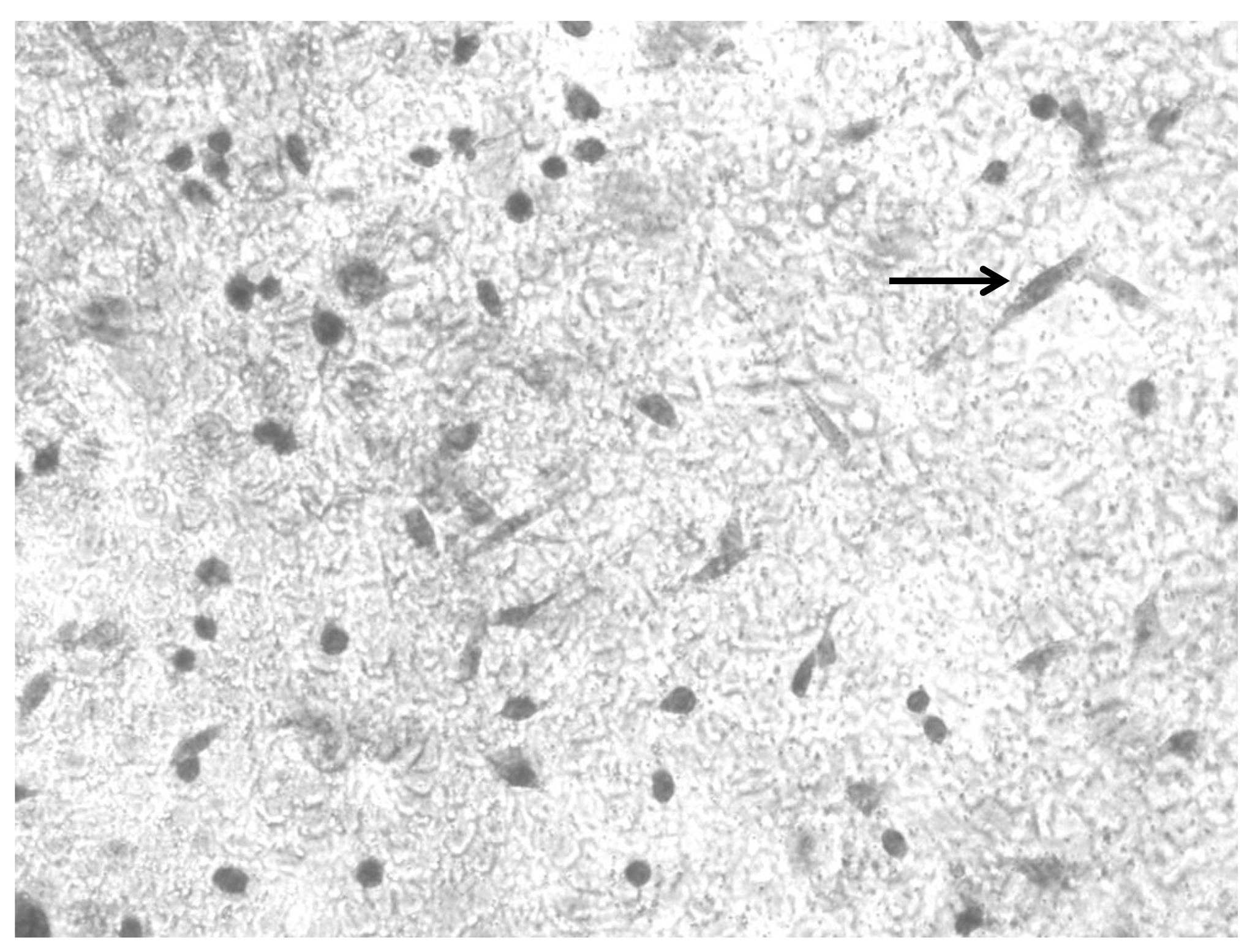

Supplement: Figure S7 — IC-21 macrophages in the co-culture. IC-21 macrophages on the bottom of the insert were fixed in 4% formaldehyde and stained with 4% crystal violet. A macrophage is identified with an arrow. (TIF) [file pone.0081104.s007.tif]
